# *Bacillus subtilis* and lactic acid bacteria improve the growth performance and blood parameters and reduce *Salmonella* infection in broilers

**DOI:** 10.14202/vetworld.2020.2663-2672

**Published:** 2020-12-16

**Authors:** Nalisa Khochamit, Surasak Siripornadulsil, Peerapol Sukon, Wilailak Siripornadulsil

**Affiliations:** 1 Department of Microbiology, Faculty of Science, Khon Kaen University, Khon Kaen 40002, Thailand; 2 Research Center for Environmental and Hazardous Substance Management, Khon Kaen University, Khon Kaen 40002, Thailand; 3 Center of Excellence on Hazardous Substance Management (HSM), Patumwan, Bangkok, 10330 Thailand; 4 Research Group for Preventive Technology in Livestock, Department of Anatomy, Faculty of Veterinary Medicine, Khon Kaen University, Khon Kaen 40002, Thailand

**Keywords:** broiler, *Bacillus subtilis*, lactic acid bacteria, probiotics

## Abstract

**Aim:**

The aim of the study was to determine the potentials and effects of *Bacillus subtilis* and lactic acid bacteria (LAB) as probiotics on broiler growth, health, and *Salmonella* infection.

**Materials and Methods:**

To evaluate the inoculum size applicable for broilers, 1-day-old broilers were orally fed fresh cultures of single strains and a *B. subtilis* KKU213/*Pediococcus pentosaceus* NP6 mixture at 10^8^ and 10^12^ colony-forming unit (CFUs)/mL/chick. The body weight gain (BWG), *Salmonella* contamination level and total *Bacillus* and LAB abundances in the crop and intestine were measured. Subsequently, 1-day-old broilers were orally fed of KKU213, CH403, and *Pediococcus acidilactici* SH8 at 10^10^ CFUs/mL, followed by inulin. After 35 days, the BWG, *Bacillus* and LAB abundances in the cecum, blood parameters, and KKU213 colonization were assessed.

**Results:**

The broilers fed single strains or KKU213+NP6 exhibited a higher BWG and a higher crop LAB abundance than the controls (p<0.05). Probiotic feeding decreased the intestinal *Salmonella* abundance and correspondingly increased the LAB abundance. The broilers fed the mixed culture (KKU213+CH403+SH8) followed by prebiotics showed lower mortality, higher blood high-density lipoprotein levels, and lower blood uric acid levels than the controls (p<0.0004). Probiotic feeding significantly increased the *Bacillus* and LAB counts (p<0.05). A CE330 isolate obtained from the cecum after 35 days of KKU213 feeding was closely related to *B. subtilis* KKU213.

**Conclusion:**

*B. subtilis* KKU213 is a potent probiotic strain that can survive, colonize and reduce *Salmonella* infection in broilers and improve their growth and health. This strain, combined with different LAB can act synergistically in the gut and promote broiler growth.

## Introduction

Broilers (*Gallus gallus domesticus*) are the most economically important widespread domestic animals for poultry meat production [1]. Farmers often use antibiotics to enhance growth and prevent pathogens in broilers, and one important example is *Salmonella* infection, a major foodborne illness called salmonellosis. Among the pathogenic *Salmonella* serovars, *Salmonella enterica* Enteritidis and Typhimurium have been implicated in foodborne gastroenteritis worldwide. Antibiotics are mostly recognized as harmful chemicals and lead to increases in antibiotic-resistant bacteria, an imbalance of the microflora in the gut, and drug residues in food products [2]. The use of probiotics as alternatives to antibiotics in the poultry industry has become an increasingly popular due to the global trend in agriculture of banning the use of in-feed antibiotics as growth promoters and can potentially minimize enteric diseases [3,4].

Probiotics are live microorganisms that benefit the health of the host by improving its nutritional and intestinal microbial balance [5,6]. The microflora in the gastrointestinal (GI) tract of poultry might be modified by probiotics, which can play a role in the competitive exclusion (CE) of pathogens, including *Salmonella*. CE is one of the modes of action through which a beneficial probiotic protects against pathogen infection in the intestinal epithelial cells of animals. However, whether effective CE through the use of a single strain or a mixture of probiotics results in activity, such as immunomodulation, adhesion site competition, and antimicrobial agent production, by one or multiple strains in the GI tract of the host remains unclear [7]. The inhibition of *Salmonella* infection by lactic acid bacteria (LAB) in intestinal epithelial cells has been documented [8,9]. Among bacterial species, several members of LAB and *Bacillus*, either as single or combined cultures, have been widely used as probiotics or microbial feed additives for chickens [10,11]. We have previously reported the potential of *Bacillus subtilis* KKU213 and some LAB strains as potential probiotics in broilers [10,12].

The aims of this study were to determine the efficiency of selected bacteriocin-producing strains of *B. subtilis* KKU213 and LAB as probiotics that can improve the growth, microbial gut community, and health of broiler and reduce *Salmonella* contamination in these organisms. The effects of different numbers of bacterial cells administered as a feed inoculum consisting of single strains or a mixture were assessed.

## Materials and Methods

### Ethical approval

All animal experiments were conducted in accordance with the guidelines and recommendations of the Institutional Animal Care and Use Committee and approved by the Animal Ethics Committee of Khon Kaen University (approval no. AEKKU 22/2558).

### Study period and location

The animal experiments were carried out at the Department of Anatomy, Faculty of Veterinary Science, Khon Kaen University, Thailand, in July-August 2018.

### Bacterial strains and culture conditions

*B. subtilis* KKU213 (KF220378), which was isolated from soil, is a bacteriocin producer and potential probiotic in broilers [12]. This strain was cultivated in *Bacillus* broth (1% peptone, 0.5% inulin, 0.5% NaCl, 0.5% beef extract, and 0.3% K_2_HPO_4_) at 50°C with shaking at 150 rpm for 24 h. The three LAB strains used in this study include (i) *Pediococcus pentosaceus* NP6 (KT00456.1), which was isolated from fermented fish and is an effective *Salmonella* inhibitor in fish samples [13]; (ii) *Pediococcus acidilactici* SH8 (MF061302.1), which was isolated from fermented shrimp, can inhibit Gram-positive and Gram-negative bacterial pathogens and does not exert a negative impact on broilers [10]; and (iii) CH403, which was isolated from the intestine of a domestic Thai fowl and can inhibit a wide range of Gram-negative bacteria. All LAB strains were cultivated in de Man, Rogosa and Sharpe (MRS) broth under microaerobic conditions at 42°C for 48 h. All 18 serovars of *S*. *enterica* were isolated from contaminated chicken meat and cultured by growing in nutrient broth and swabbed on Salmonella Shigella (SS) agar when tested for anti-*Salmonella* activity.

### *In vitro* anti-*Salmonella* activity of *P. pentosaceus* NP6

*P. pentosaceus* NP6 was grown in MRS broth and then incubated at 37°C for 48 h. The cell-free supernatant (CFS) was centrifuged at 8000 rpm for 30 min, filtered through a 0.22-μm filter and lyophilized. The protein concentration of CFS was measured using the Quick Start™ Bradford Protein Assay (Bio-Rad Laboratories, Hercules, USA). The CFS was resuspended in 1 mL of ddH_2_O, heated at 100°C for 15 min and 121°C for 15 min, and treated with 10 mg/mL proteolytic enzymes, trypsin, and pepsin for 3 h. Subsequently, 10 μL of the treated CFS samples was dropped on the swabbed culture of 18 serovars of *S. enterica*, and the cultures were incubated at 37°C for 18-24 h.

### Effect of bacterial inocula and mixed cultures on the growth of and *Salmonella* contamination in broilers

*B. subtilis* KKU213 was cultured in LB broth and incubated at 42°C for 24 h, and *P. pentosaceus* NP6 was cultured in MRS broth and incubated at 42°C for 24 h. The bacterial cells were centrifuged, washed with sterile 0.85% NaCl and adjusted to 10^8^ and 10^12^ colony-forming units (CFUs)/mL. One milliliter of each culture was orally fed to 1-day-old broilers obtained from a commercial broiler hatchery.

#### Husbandry, diets, experimental design, and viable cell count

A total of 105 1-day-old Cobb broiler chickens were used in this experiment. The chickens were randomly allocated to seven groups, each of which included 15 birds. The chickens in all the groups were orally fed as follows: the birds belonging to the control group were fed sterile 0.85% (w/v) NaCl, and those in the probiotic-fed groups were fed KKU213, NP6, or KKU213+NP6 at doses of either 10^8^ or 10^12^ CFUs/mL. This oral feeding was performed before the chickens were allowed access to food or water. The birds were allowed *ad libitum* access to the diets and water throughout the experimental period and weighed weekly, and their mortality was recorded on a daily basis. The body weight gain (BWG) was calculated as the difference between the final and initial bird weights during each weighing period. After 1, 6, and 18 days, five chickens from each group were sacrificed, and the bacteria were aseptically swabbed from the crop and the intestine (jejunum) and resuspended in 0.85% NaCl. The samples were spread on *Bacillus* agar for *Bacillus* spp., MRS agar for LAB, and SS agar for *Salmonella* spp. and incubated at 50°C, 42°C, and 37°C, respectively, to determine the number of viable cells (CFUs/mL).

### Assessment of the antibiotic susceptibility of selected isolates from bacteria-fed groups

*B*. *subtilis* KKU213 and the selected isolates from the crop and intestine of broilers fed KKU213 were grown in *Bacillus* broth and incubated at 50°C for 18 h. The cells were adjusted to the McFarland 0.5 scale and swabbed on LB agar. Disks of the selected antibiotics, including chloramphenicol (C30), lincomycin (MY2), colistin (CT10), and oxytetracycline (OT30), were placed on the bacterial swabs, and the swabs were then incubated at 37°C for 24 h. The clear zones surrounding the antibiotic disks were measured.

### Molecular identification by *16S rRNA* sequencing

The isolates from the crops and intestines showing the same antibiotic susceptibility as KKU213 were later identified by polymerase chain reaction (PCR), and the *16S* *rRNA* gene was sequenced using the 20F (5′-GAG TTT GAT CCT GGC TCA G-3′) and 1500R (5′-GTT ACC TTG TTA CGA CTT-3′) primers. The PCR program was as follows: 94°C for 3 min; 34 cycles of denaturation at 94°C for 1 min, annealing at 48°C for 1 min, and polymerization at 72°C for 2 min; and a final extension of 5 min at 72°C.

### Effect of the probiotic mixtures on the growth, blood parameters, and gut microbes of broilers

*B. subtilis* KKU213 was prepared as previously described. CH403 and *P. acidilactici* SH8 were cultivated in MRS broth under microaerobic conditions at 42°C for 48 h. The bacterial cells were centrifuged, washed with sterile 0.85% NaCl, and adjusted to 10^10^ CFUs/mL. One milliliter of each culture was orally fed to 1-day-old broiler chickens obtained from a commercial broiler hatchery. This experiment was performed using the same approach previously reported by Khochamit *et al*. [12].

#### Husbandry, diets, experimental design, and viable cell count

A total of 68 Cobb broilers at 1 day of age were used and divided into two groups, and each group included 34 birds. The broilers allocated to the control group were orally fed 1 mL of sterile 0.85% (w/v) NaCl, and those belonging to the experimental group were fed the KKU213+SH8+CH403 mixture at a dose of 10^10^ CFUs/mL. The broilers were fed twice on the 1^st^ and 3^rd^ days and allowed *ad libitum* access to the diets and water. On the 5^th^ day, 1 mL of 2% (w/v) inulin was orally fed to the bacteria-fed group, and starting on the 21^st^ day; the broilers were fed 1% (w/v) inulin for 1 week. The chickens were weighed every 2 days, and their mortality was recorded on a daily basis. After 1, 10, 20, and 30 days, the bacterial number was determined by obtaining aseptic swabs from the cloaca. The samples were resuspended in 0.85% NaCl, spread, incubated on *Bacillus* agar at 50°C for *Bacillus* spp. and on MRS agar at 42°C for LAB for 24-48 h, and counted. On the 35^th^ day, all the chickens were sacrificed, and blood was collected. The levels of cholesterol, triglyceride, high- and low-density lipoproteins (HDLs and LDLs), and uric acid in the blood were measured. The ceca were swabbed, and the swabs were resuspended in 0.85% NaCl, serially diluted, spread, and counted on *Bacillus* agar for *Bacillus* spp. and MRS agar for LAB as previously described.

#### Antibacterial activity of selected isolates from the bacteria-fed group against Bacillus cereus

The antibacterial activity against *B. cereus* ATCC 11778 of *B. subtilis* KKU213 and selected isolates from the cecum and cloacal swabs was tested. The isolates were grown on LB broth, incubated at 37°C for 24 h and streaked on *B. cereus*-swabbed plates. The plates were incubated at 37°C for 24 h, and the clear zones around each colony were measured.

#### Molecular identification of anti-B. cereus isolates

Three milliliters of a select bacterial culture incubated for 18 h was centrifuged at 13,000 rpm for 2 min. DNA was extracted using the phenol:chloroform:isoamyl method. Identification was performed using primers targeting *16S* rDNA: 20F (5′-GAG TTT GAT CCT GGC TCA G-3′) and 1500R (5′-GTT ACC TTG TTA CGA CTT-3′). The subtilosin A genes were also identified using the specific primers sboA-F (5′-CAG AGC TCA TGA AAA AAG CTG TCA TTG TAG AAA AC-3′), sboX-F (5′-ATG AGC TCG TGT TCT TCA TAA GAT AGA TA-3′), albA-F (5′-TTG AAT TCT TGT TTA TAG AGC AGA TGT TTC CAT TT-3′), and albA-R (5′-GTG CGG CCG CAC GTA CTT CGC CGA ACG GGC TG-3′) [14]. PCR was performed under the following conditions: 94°C for 3 min; 34 cycles of denaturation at 94°C for 30 s, annealing at 55°C for 30 s (for the *16S* *rRNA* gene) or 62°C for 30 s (for subtilosin A genes), and polymerization at 72°C for 90 s; and a final extension at 72°C for 3 min. The PCR products were subjected to DNA sequencing. Phylogenic trees and the evolutionary distances of the *16S* *rRNA* sequences were calculated using the neighbor-joining model and the maximum likelihood functions of Molecular Evolutionary Genetics Analysis (MEGA) version 5.0, which is licensed as a proprietary freeware [15].

### Statistical analysis

One-way analysis of variance was used to analyze the data obtained from these experiments. If the main effect was found to be significant at p<0.05, the differences between means were analyzed by Tukey’s honest significant difference test.

## Results

### *In vitro* antibacterial activity of *P. pentosaceus* NP6 against *Salmonella*

The total protein concentrations of the lyophilized CFS of NP6 before and after heating were 1533.33 and 1700 μg/mL, respectively. The lyophilized CFS of *P. pentosaceus* NP6 showed a similar level of antibacterial activity against 18 serovars of *S*. *enterica* before and after heating at 100 and 121°C or after treatment with proteolytic enzymes. The exception was the inhibition of *S*. Emek 4759: The NP6-CFS samples subjected to the various treatments showed different anti-*Salmonella* activities, and those heated at 121°C for 15 min exhibited the highest activity (Table-1).

**Table-1:** Antibacterial activity against *Salmonella enterica* strains isolated from contaminated chicken meat of the lyophilized cell-free supernatant of *Pediococcus pentosaceus* NP6 heated at 100°C for 15 min or at 121°C for 15 min or treated with pepsin or trypsin.

*Salmonella* serovar	Inhibition zone (mm)						

	Control	100°C, 15 min	121°C, 15 min	1 mg/mL Pepsin, 3 h	1 mg/mL Trypsin, 3 h	SEM	p-value
*Salmonella* Agona 1935	9.69	8.75	9.38	10.13	9.50	0.76	0.78
*Salmonella* Amsterdam 1936	9.38	8.88	9.38	10.25	9.13	0.33	0.09
*Salmonella* Braenderup	10.75	9.75	11.50	10.38	9.75	0.82	0.53
*Salmonella* Bareilly 5387	10.63	9.00	9.75	11.00	10.00	0.45	0.05
*Salmonella* Brunei 916	9.13	8.63	9.00	9.50	9.25	0.65	0.91
*Salmonella* Emek 4759	9.75^bc^	9.25^bc^	11.50^a^	10.25^ab^	8.50^c^	0.31	<.0001
*Salmonella* Enteritidis	9.25	8.88	9.75	9.75	8.50	0.39	0.14
*Salmonella* Enteritidis 176-1	9.00	9.06	9.38	8.88	8.50	0.22	0.14
*Salmonella* Hvittingfoss 5382	10.00	9.56	8.75	8.75	9. 00	0.45	0.25
*Salmonella* Kentucky	10.38	9.94	10.75	9.00	9.00	0.47	0.06
*Salmonella* Mbandaka 5412	9.56	9.50	10.13	9.13	9.00	0.34	0.21
*Salmonella* Ohio	10.13	9.88	10.50	9.25	9.00	0.36	0.05
*Salmonella* Paratyphi 8486	12.38	11.93	10.00	10.50	10.75	0.60	0.06
*Salmonella* Singapore 5416	10.31	10.00	9.50	8.00	9.00	0.48	0.03
*Salmonella* Typhimurium	11.00	9.50	9.50	10.50	10.38	0.56	0.28
*Salmonella* Typhimurium 5313	8.88	8.94	9.75	8.63	8.63	0.37	0.24
*Salmonella* Urbana 5381	10.13	8.75	9.38	9.25	9.75	0.38	0.17
*Salmonella* Weltevreden 5383	11.06	9.63	10.25	10.25	9.75	0.42	0.18

### Effects of *B. subtilis* KKU213 and *P. pentosaceus* NP6 on the bacterial number in broilers

**Figure-1:**
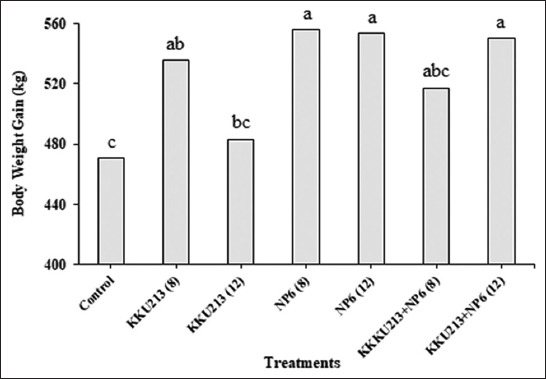
Body weight gain of broilers on 18 days of age after feeding KKU213 and/or NP6 at 10^8^ (8) and 10^12^ (12) colony-forming units/mL.^a,b,c^ Means with different superscripts within a column are significantly different (p<0.05). n=15.

**Figure-2:**
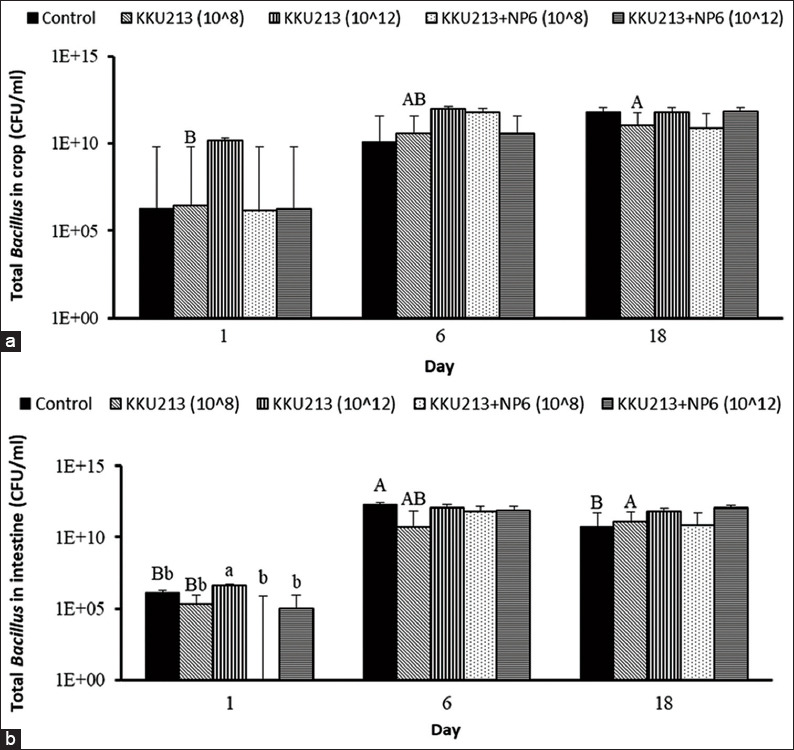
Viable count of total Bacillus in the (a) crop and (b) intestine of broilers that were orally fed KKU213 and KKU213+NP6 for 1, 6, and 18 days. The bars represent the means±SEMs at p<0.05. n=5.^A,B,C^ Means show significant differences within the same group at different times.^a,b,c^ Means show significant differences between groups on the same day.

**Figure-3:**
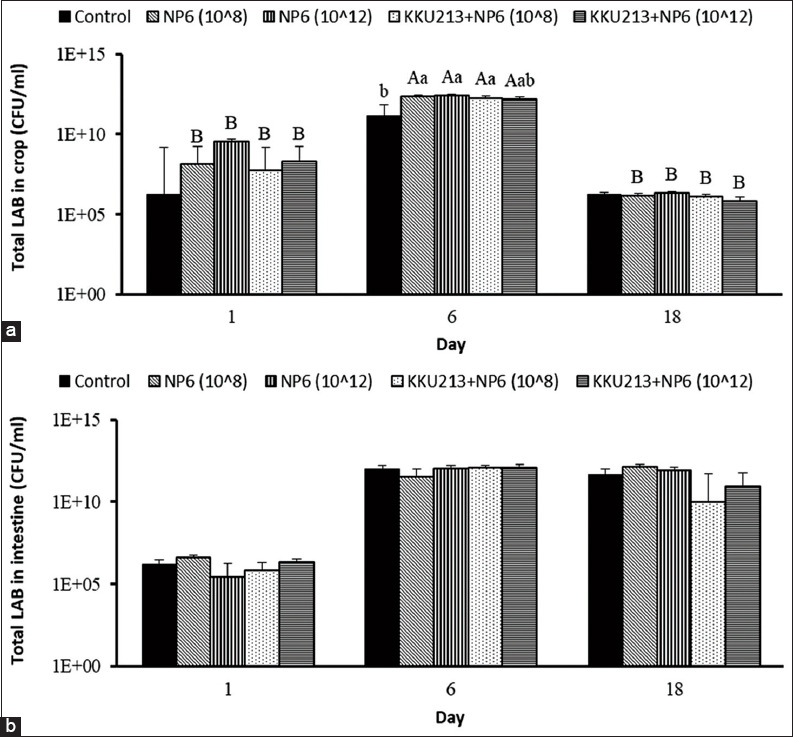
Viable count of total LAB in the (a) crop and (b) intestine of broilers that were orally fed NP6 and KKU213+NP6 for 1, 6, and 18 days. The bars represent the means±SEMs at p<0.05.^A,B,C^ Means show significant differences within the same group at different times.^a,b,c^ Means show significant differences between groups on the same day.

**Figure-4:**
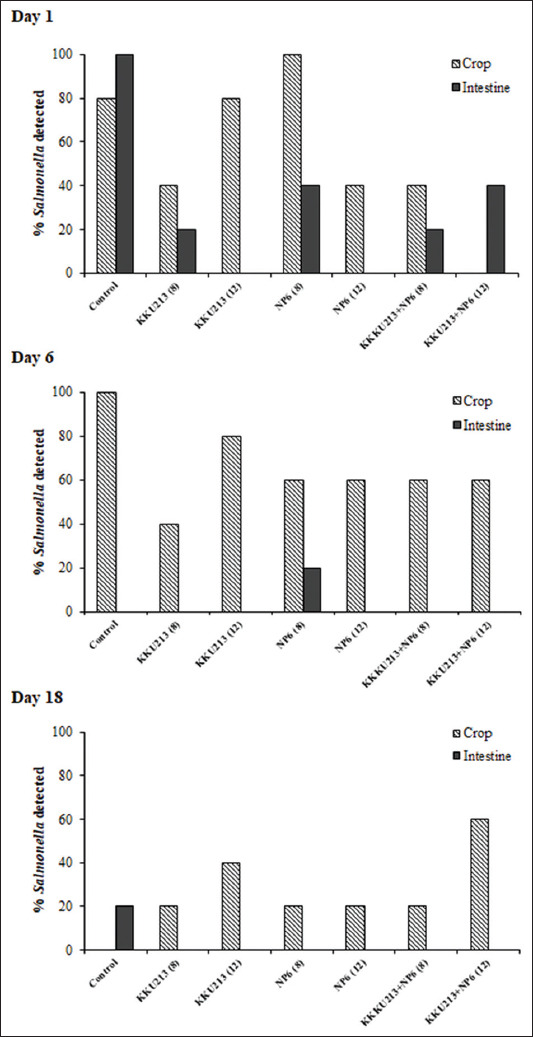
Detection of *Salmonella* in the crop and intestine (jejunum) of broilers that were orally fed *Bacillus subtilis* KKU213 and/or *Pediococcus pentosaceus* NP6 for 1, 6, and 18 days.

Broilers were fed KKU213, NP6, and KKU213+NP6 at inoculum sizes of 10^8^ and 10^12^ CFUs/mL, the effects were evaluated. Four out of six groups of chickens fed bacteria exhibited a higher BWG at day 18 than the controls (Figure-1). The percent mortality of all the groups was as low as 0 or 6.7. The numbers of *Bacillus* in the KKU213-fed group and those of LAB in the NP6-fed groups in the crop and intestine were investigated. The numbers of *Bacillus* in the crop and intestine of the broilers belonging to the KKU213-10^8^ group were significantly increased at day 18 compared to the numbers on day 1, whereas the numbers in the intestine from day 6 to 18 were decreased in the control broilers (Figure-2). On day 1, the highest number of *Bacillus* in the intestine was found in the broilers belonging to the KKU213-10^12^ group. In all the NP6-fed groups, the numbers of LAB in the crop were significantly increased at day 6 and decreased at day 18 (Figure-3). On day 6, the number of LAB in all the NP6-fed groups was higher than that in the control group. In the intestine, the number of LAB was increased on day 6 and remained at a similar level until day 18. However, the LAB number in the intestine did not differ among the groups at the same time or among time points within the same group. The detection of *Salmonella* showed a relatively higher abundance in the crop than in the intestine. The control group exhibited the highest *Salmonella* abundance in the crop and intestine on days 1 and 6. Compared with the 20% level observed in the control group on day 18, no *Salmonella* was detected in the intestine of the broilers belonging to all bacteria-fed groups at the same time point (Figure-4).

### Antimicrobial susceptibility and identification of *Bacillus* isolates from KKU213-fed broilers

**Figure-5:**
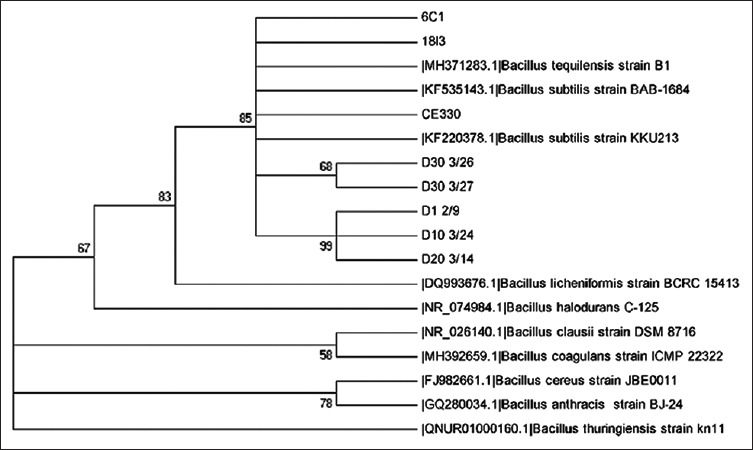
Phylogenetic tree of partial *16S rRNA* nucleotide sequences of the selected isolates 6C1, 18I3, D1 2/9, D10 3/24, D20 3/14, D30 3/26, D30 3/27, and CE330 derived from KKU213-fed broilers in comparison to *Bacillus subtilis* KKU213.

A total of 21 bacterial isolates from the GI tract of KKU213-fed broilers were tested for their antibiotic susceptibility, and two isolates showed a similar profile to *B. subtilis* KKU213. 6C1 was isolated from the crop on day 6, and 18I3 was isolated from the intestine on day 18. These two isolates were analyzed by partial *16S* *rRNA* sequencing. The 6C1 (798 bp) and 18I3 (1381 bp) sequences shared 99% and 100% identity with the *B. subtilis* KKU213 sequence, respectively. Phylogenetic analysis revealed that these isolates were closely related to *B. subtilis* KKU213, *B. subtilis* subsp. inaquosorum (HG008722.1) and *Bacillus tequilensis* S2Y2-a (JQ828865) (Figure-5).

### Effects of the probiotic mixtures on the growth performance and blood parameters of broilers

On day 35, the broilers belonging to the KKU213+CH403+SH8-fed group exhibited a lower BWG but markedly lower mortality than the control broilers (Table-2). The analyses of the blood parameters revealed that the cholesterol (p<0.0001), HDL (p=0.001), and LDL (*p* = 0.0003) levels were 17.10, 14.53, and 20.83% higher in the mixed culture-fed group than in the control group, respectively. In addition, the administration of the mixed culture significantly decreased the uric acid level by 63.40% (p=0.0004) but has no significant effect on the triglyceride levels (Table-2).

**Table-2:** Effect of a mixed culture of *Bacillus subtilis* KKU213 and LAB on performance and blood parameters of broilers.

Group	Performance		Blood parameters (mg/dL)				
	
	BWG	% mortality	CHL	TG	HDL	LDL	Uric acid
Control	1663.72^a^	14.71^a^	129.50^b^	21.72	73.42^b^	51.74^b^	3.17^a^
KKU213+CH403+SH8	1589.76^b^	5.88^b^	151.65^a^	25.20	84.09^a^	62.52^a^	1.94^b^
SEM	0.006	0.004	2.88	1.71	1.95	17.76	0.21
p-value	<0.0001	<0.001	<0.0001	0.184	0.001	0.0003	0.0004

CHL=cholesterol, TG=triglyceride, HDL=high-density lipoprotein, LDL=low-density lipoprotein.^a,b^ Means with different superscripts within a column are significantly different (p<0.05). n=34

### Effects of the mixed cultures on the gut microbes and colonization of KKU213 in broilers

On day 30 after feeding, the number of *Bacillus* in cloaca samples swabbed from the mixed culture-fed group was significantly (p<0.0001) higher than that found in the samples obtained from the control broilers. In addition, the LAB number found in the cloaca swabs from the mixed culture-fed group on 20 and 30 days after feeding was higher than that found in the samples from the control group (Table-3). A total of 24 bacterial isolates from cloaca swabs and cecum samples from bacteria-fed broilers were screened for antibacterial activity against *B. cereus* relative to that of *B. subtilis* KKU213, and six isolates (CE330, D30 3/26, D30 3/27, D1 2/9, D10 3.24, and D20 3/14) were selected for partial *16S* *rRNA* sequence analysis. CE330, D30 3/26, and D30 3/27 were found to be closely related to *B. subtilis* KKU213 (KF220378.1), *B. subtilis* BAB-1684 (KF535143.1), and *B. tequilensis* B1 (MH371283), respectively. Among these three strains, CE330, which was isolated from the cecum, shared the highest sequence identity with the KKU213 sequence (Figure-5). In addition, the *sboA-sboX* genes encoding subtilosin A in KKU213 were also detected in CE330, D30 3/26, and D30 3/27, and these strains shared 100% identity with SboA and SboX amino acid sequences [12].

**Table-3:** Effects of a mixed culture on the total *Bacillus* and lactic acid bacteria in the cloaca and cecum of broiler chickens.

Group	Total *Bacillus* (CFUs/mL)					Total lactic acid bacteria (CFUs/mL)				
	
	Cloaca swab				Cecum D35	Cloaca swab				Cecum D35
	
	D1	D10	D20	D30		D1	D10	D20	D30	
Control	1.91×10^8^	2.62×10^6^	5.41×10^5^	5.32×10^6b^	3.29×10^9^	6.00×10^6^	5.63×10^6^	2.17×10^5b^	1.19×10^5b^	5.27×10^9^
KKU213+CH403+SH8	1.97×10^7^	3.89×10^6^	4.01×10^6^	7.11×10^7a^	3.24×10^9^	8.93×10^6^	1.67×10^6^	9.57×10^5a^	1.51×10^6a^	4.04×10^9^
SEM	1.01×10^8^	1.03×10^6^	1.13×10^6^	1.05×10^7^	7.20×10^8^	2.89×10^6^	3.13×10^6^	1.69×10^5^	2.29×10^5^	1.32×10^9^
p-value	0.274	0.392	0.085	<0.0001	0.962	0.769	0.397	0.004	0.0001	0.643

D=Day; ^a,b^Means within a column are significantly different (p<0.05). n=34

## Discussion

The potential of probiotic candidates depends on several factors, such as viable cell numbers, acid and bile salt tolerance, production of antimicrobial metabolites, and gut colonization. We isolated *Bacillus* and LAB bacteria from several sources based on the described properties. Among the selected isolates, the bacteriocin-producing strain *B. subtilis* KKU213 has exhibited interesting potential due to its effects on promoting the growth of broilers [10,12]. *P. pentosaceus* NP6 inhibits several *Salmonella* serovars *in vitro* and in food matrices [13], but KKU213 does not exhibit this property. The inhibition of *Salmonella* by NP6 was maintained even if the CFS was heated or treated with proteolytic enzymes, which suggests that this strain likely produces some heat-resistant bacteriocin because this sample contains a relatively high amount of total soluble protein. Some *P. pentosaceus* strains can produce pediocin, which mostly inhibits Gram-positive bacteria and is sensitive to heat [14], but pediocin-encoding genes were not detected in NP6 (unpublished data). Thus, other bacteriocins are likely responsible for the inhibition of *Salmonella* observed in this study. The increase in soluble protein content of the autoclaved CFS of NP6 suggested that some proteins were likely degraded into small and soluble proteins by the associated high pressure and heat.

*Salmonella* contamination is a serious problem in poultry production. To further investigate the probiotic properties of KKU213, the effects of its combination with the anti-*Salmonella* NP6 strain were evaluated in broilers. Moreover, antagonistic activity was not observed between these bacteria. To determine the optimal dose and effectiveness of a probiotic inoculum for broilers, sterile 1-day-old broilers were fed the probiotics at two different dosages, 10^8^ and 10^12^ CFUs/mL as either single or mixed feeding. The mixed culture increased the BWG of the four bacteria-fed groups, which indicated that the combination of *Bacillus* and LAB improved the growth of broilers, with the exceptions of KKU213 at 10^12^ CFUs/mL, which might be a too-high dose as a single strain, and of KKU213+NP6 at 10^8^ CFUs/mL which might be too low dose as the mixed strain (Figure-1). The chickens were raised under standard and appropriate conditions, as demonstrated by their low mortality rates (0 or 6.7%).

The comparison of the effects of *B. subtilis* KKU213 and/or *P. pentosaceus* NP6 revealed similar impacts in broilers and resulted in similar viable cell counts. Both KKU213 and NP6 were highly resistant to an acidic pH value of 2 [12,13], and thus, these strains can likely tolerate the low pH in the stomach of broilers, can multiply in this environment and facilitate the growth of indigenous *Bacillus* and LAB in the crop and jejunum (Figures-2 and 3), which is consistent with previously observed results [10,12]. In general, similar microbial profiles were found among all probiotic-fed groups and the control. However, the feeding of broilers with 10^8^ CFUs of KKU213 resulted in the most variable number of *Bacillus* compared with the other groups (Figure-2). Feeding with 10^8^ and 10^12^ CFUs of NP6 significantly increased the number of total LAB in the crop on day 6 to a value higher than that found in the control broilers (Figure-3). The reduction in LAB detected in the crop on day 18 might be related to the low level of *Salmonella* in the crop; in contrast, the presence of *Salmonella* was not observed in the intestine (jejunum) (Figure-4), where the number of LAB was relatively higher than that in the crop (Figures-2 and 3).

According to the health parameters in this study, the administration of a probiotic strain alone or in a mixture for 6 and 18 days reduces the abundance of *Salmonella* in the intestine of broilers. On day 6, the number of LAB in the crop of the bacteria-fed broilers was also higher than that in the control broilers (Figure-3). The levels of *Salmonella* were lower in the probiotic group than in the control group, and the lowest levels were detected after 18 days (Table-1). All the described results indicate that the probiotics exert effective short-term effects on *Salmonella* in broilers. This study also revealed that NP6 inhibited several *Salmonella* serovars *in vitro*. The effect of *B. subtilis* NC11 on reducing the number of *S*. Enteritidis and the adherence of LAB to the intestinal epithelial cells of the host might result in the CE of pathogenic bacterial adhesion [16,17]. *Lactobacillus reuteri* S5 isolated from chicken feces has potential as a biocontrol agent for *S*. Enteritidis infection [18]. Although KKU213 exhibited no *in vitro* activity against *Salmonella*, the occurrence of CE might be induced by the colonization of KKU213 and/or the inhibition of *Salmonella* by NP6. In addition, some bacteriocins produced by KKU213 and NP6 could help balance the gut microbes in favor of effective microbes [12]. Overall, probiotics did not modify the bacterial profiles of *Bacillus* and LAB in the crop and intestine, which indicated that the probiotics did not negatively affect the normal flora of broilers.

Due to its significant potential as a probiotic strain, the stability and application of KKU213 in broilers were further investigated. This strain was mixed with two additional LAB strains isolated from the Thai fowl intestine (CH403, able to inhibit a wide range of Gram-negative bacteria) and from fermented shrimp (SH8, able to inhibit both Gram-positive and Gram-negative bacteria, including *Salmonella*) [10]. All three strains were able to utilize inulin (prebiotic) as a sole carbon source, and none showed antagonistic activity against each other *in vitro*. The inoculum size of 10^10^ CFUs/mL was selected because no significant difference was observed between the doses of 10^8^ and 10^12^ CFUs in the previous experiment.

The broilers fed the mixed culture (KKU213+CH403+SH8) exhibited better growth performance than those fed the control. Although their BWG was lower, the mixed culture-fed broilers were healthier and exhibited a lower mortality rate than the control broilers. Regarding blood health parameters, a significant increase in the HDL levels and a decrease in the uric acid levels (Table-2) were found in the mixed culture-fed broilers compared with the control broilers, and these changes increase the quality and nutritional value of chicken meat. Although the cholesterol levels of the mixed culture-fed broilers were higher, the level of triglycerides did not differ between the probiotic-fed and control groups. This result is consistent with the results obtained with the combination of KKU213 and four other LAB strains, which also resulted in increased HDL levels and decreased uric acid levels in broilers [10,19]. Sugiharto *et al*. (2018) reported that the lower level of serum uric acid in birds supplemented with 0.5% multistrain probiotics was associated with higher retention of protein in the body of birds [20]. The total numbers of *Bacillus* on days 30 and of LAB on days 20 and 30 detected in cloaca swabs from KKU213+CH403+SH8-fed broilers were significantly higher than those found in the swabs from the control group, which suggested that the probiotics altered the microbial gut population in a positive manner.

The feeding of probiotics combined with inulin might facilitate their multiplication and thus increase the abundance of gut microbes in the fed broilers. The combined administration also enhanced nutrient adsorption in broilers, possibly through the secretion of extracellular enzymes by *B. subtilis* KKU213 [12]. It is likely that soil-originated KKU213 was able to colonize and multiply in the broiler gut, which is not surprising because chickens usually ingest feed contaminated with outside microbes. This finding is consistent with a study reporting that spore-producing *Bacillus* are common in the soil but able to germinate in and colonize the animal gut [21].

To determine the ability of KKU213 to colonize the broiler gut after being fed to broilers, *Bacillus* isolates with similar properties to KKU213 were selected and tested. In the first experiment, two out of 21 isolates exhibited similar antibiotic susceptibility to KKU213 after 6 and 18 days. In the second experiment, six out of 24 isolates showed inhibitory activity against *B. cereus* activity after 35 days similar to that of KKU213. Among these isolates, the CE330 isolate exhibited the highest *16S* *rRNA* sequence similarity to KKU213. The results suggest that KKU213 has the potential to survive in and colonize the broiler gut. Among the vast diversity of gut microbiomes, *Bacillus* and LAB are common in the animal gut and produce several metabolites, at least 800 and 100 compounds, respectively [22], and these bacteria are considered the most important microbes that colonize the gut. Both *Bacillus* and LAB can exert a synergistic effect on their hosts through interactions with the host immune system and produce several metabolites that enhance growth and act as natural guards against some pathogens [23].

## Conclusion

The inclusive screening of probiotics from various sources and their combinations revealed the effective tools for the identification of potential probiotics in animals. The CE330 isolate derived from *B. subtilis* KKU213-fed broilers will be investigated to determine its potential as a potent probiotic strain. The application of probiotics as feed supplements in granule form instead of fresh culture and the feeding of potent probiotics originating from broilers instead of soil together with prebiotics should be further explored in poultry.

## Authors’ Contributions

WS was responsible for the research concept, data analysis, and approving the manuscript. NK conducted the research, collected and analyzed the data, and drafted the manuscript. SS advised on the experimental design and approved the manuscript. PS advised on the experimental design and the animal experiments. All the authors read and approved the final manuscript.
